# Molecular targeting in acute myeloid leukemia

**DOI:** 10.1186/s12967-017-1281-x

**Published:** 2017-08-29

**Authors:** Seah H. Lim, Patrycja M. Dubielecka, Vikram M. Raghunathan

**Affiliations:** Division of Hematology and Oncology, Brown University Warren Alpert Medical School, Rhode Island Hospital, 593 Eddy Street, Providence, RI 02903 USA

## Abstract

Acute myeloid leukemia (AML) is a heterogenous disease associated with distinct genetic and molecular abnormalities. Somatic mutations result in dysregulation of intracellular signaling pathways, epigenetics, and apoptosis of the leukemia cells. Understanding the basis for the dysregulated processes provides the platform for the design of novel targeted therapy for AML patients. The effort to devise new targeted therapy has been helped by recent advances in methods for high-throughput genomic screening and the availability of computer-assisted techniques for the design of novel agents that are predicted to specifically inhibit the mutant molecules involved in these intracellular events. In this review, we will provide the scientific basis for targeting the dysregulated molecular mechanisms and discuss the agents currently being investigated, alone or in combination with chemotherapy, for treating patients with AML. Successes in molecular targeting will ultimately change the treatment paradigm for the disease.

## Background

Despite the advance of modern chemotherapy, the prognosis of patients with acute myeloid leukemia (AML) has remained poor and little progress has been made that improves long term outcome of these patients. For more than four decades since the combination of an anthracycline and cytarabine was first used for induction therapy, the “3 + 7” regimen has remained the standard therapy for AML. The long term disease-free survival of AML patients under age 60 remains around 40% [1], with minimal improvement over the past several decades, suggesting that the gains from conventional chemotherapy may have been maximized. New approaches are, therefore, needed if further improvement in the outcome for AML patients is desired.

AML is a clonal malignancy associated with a wide-spectrum of genetic alterations. In addition to well-described chromosomal abnormalities, a multitude of mutations occur and they contribute to AML pathogenesis, either due to their effects of tumor suppressor genes or as drivers of intracellular oncologic signaling pathways or modifiers of epigenetics. The magnitude and frequency of these abnormalities, and their pathologic implications, were not fully appreciated until the last decade as novel techniques for the analysis of whole genome sequencing have become available.

The molecular events associated with AML have long been used to predict prognosis [2]. With an expanding understanding of the molecular genetic alterations underlying AML pathogenesis, recent efforts have concentrated on specific targeting of intracellular events driven by these abnormal proteins. Molecular targeting is a particularly attractive therapeutic approach for several reasons. First, the therapeutic efficacy of molecular targeting may complement the benefits provided by conventional chemotherapy. Second, the approach may be more specific to each patient’s molecular landscape and minimize systemic toxicity. Third, it may offer an increased likelihood of eradication of the malignant clones that drive the disease and often being responsible for disease relapse.

Here we will review the intracellular mechanisms and pathways that provide the platforms for molecular targeting in AML. Specifically, we will discuss therapies targeting FMS-like tyrosine kinase 3 (FLT3) and pathways associated with DNA methyltransferase (DNMT)3A, ten-eleven-translocation (TET)2, and IDH (isocitrate dehydrogenase) 1/2. We will also summarize the current status of utilizing histone deacetylase (HDAC), bromodomain and extra terminal (BET), and disruptor of telomeric silencing 1-like (DOT1L) inhibitors in AML. Finally, we will discuss the role of therapies targeting the anti-apoptotic protein, BCL (B-cell lymphoma)-2, as it has recently been shown that IDH1/2 mutation status may identify patients who are most likely to respond to therapeutic inhibition of BCL-2 [3]. Since molecular therapy of the promyelocytic leukemia-retinoic acid receptor alpha (PML-RARα) in acute promyelocytic leukemia (APL) is well-established, we will limit our review to novel agents for non-APL AML. This review is not meant to be an exhaustive discussion of all emerging agents. Instead we will summarize the results of some of the clinical studies carried out so far.

## Main text

### Targeting FLT3 signaling pathway

#### FLT3 mutations

**Fig. 1:**
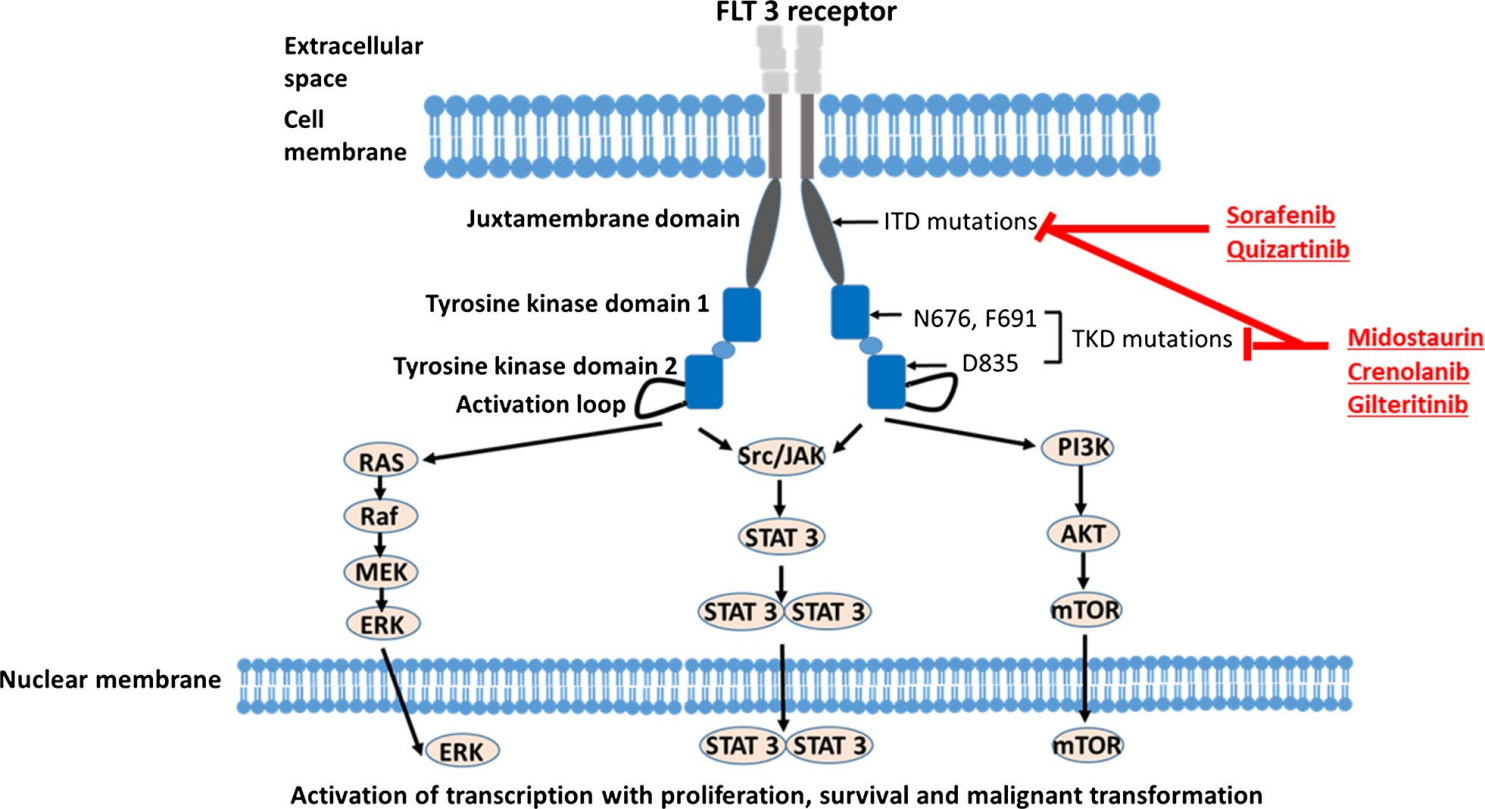
FLT3 kinase signaling pathway and sites blocked by FLT3 inhibitors. Sorafenib and quizartinib inhibit only FLT3–ITD mutations, while midostaurin, crenolanib, and gilteritinib inhibit both FLT3–ITD and FLT3 TKD mutations

FLT3 is a surface receptor that consists of an extracellular ligand-binding domain, a transmembrane domain, a juxtamembrane domain, and two tyrosine kinase domains. Engagement of the wildtype receptor with the FLT3 ligand triggers a cascade of downstream events that signal cell proliferation [4, 5]. This is achieved first through autophosphorylation of the tyrosine residues on the receptor and then by the consequent phosphorylation and activation of the RAS, Src/JAK (Janus kinase), and PI3K pathways (Fig. 1). High levels of the downstream effector of the RAS pathway, ETS2, have recently been found to predict for poorer prognosis [6].

Mutations of the FLT3 receptor occur in nearly one-third of patients with AML and are one of the most frequent mutations encountered in this disease [7]. The mutations occur either as internal tandem duplications (FLT3/ITD mutations) in or near the juxtamembrane domain, or as point mutations that result in single amino acid substitutions within the activation loop of the tyrosine kinase domain (FLT3/TKD mutations). FLT3/ITD mutations occur in 24% [8] and FLT3/TKD mutations in 7% of AML [9]. Patients with FLT3/ITD mutations typically have high white cell counts at disease presentation and have normal or intermediate risk karyotypes. Although the likelihood of attaining a complete remission (CR) of the disease is similar to other AML patients, the duration of remission is usually short and the relapse rate high. FLT3/TKD mutations tend to confer slightly better prognosis. Interestingly, FLT3 phosphorylation has also been observed in a large proportion of AML patients, even in the absence of FLT3 mutations [9, 10].

FLT3 mutations result in a constitutively active kinase [10]. It addition to mediating the intracellular signaling events observed when wild type FLT3 receptor interacts with its ligand, FLT3/ITD activates the Stat 5 pathway [11–14] and upregulates the serine threonine kinase, Pim-1/2 [13, 15]. Both these processes promote leukemia cell proliferation and mediate anti-apoptotic effects. FLT3/ITD mutations also promote genomic instability by inducing the production of reactive oxygen species (ROS) that enhance DNA double-strand breaks and repair errors [16].

#### FLT3 inhibitors for AML

Based on the frequent occurrence of FLT3 mutations and the poor clinical outcome in patients harboring the mutations, molecular targeting of FLT3 kinase is an attractive therapeutic option for AML. Since the identification of FLT3 mutations, several molecular agents have been developed to target the FLT3 kinase. These include sorafenib and quizartinib that inhibit FLT3/ITD mutant receptor and midostaurin, crenolanib, and gilteritinib that inhibit both FLT3/ITD and FLT3/TKD mutant receptors (Fig. 1). Most of these agents are multi-kinase inhibitors.

##### Sorafenib

Sorafenib has been used off-label in the treatment of relapsed/refractory AML. It is an oral agent that is 1000–3000 times more potent in inducing growth inhibition and apoptosis in AML cells that harbor FLT3/ITD or D835G mutations than in those that harbor D385Y mutation or wildtype FLT3 kinase [17]. It is a multi-kinase inhibitor that also has activity against KIT, vascular endothelial growth factor receptor (VEGFR), and platelet-derived growth factor receptor (PDGFR). In a phase I study, 16 patients with relapsed/refractory AML were randomly assigned to receive sorafenib in 21-day cycles of 5 days per week (n = 7 patients) or of 14 days (n = 9 patients). In both arms, the starting dose level was 200 mg twice daily. Subsequent dose levels were 600, 800, and 1200 mg daily in cohorts of three subjects at each dose level. Leukemic burden was reduced in patients with FLT3/ITD mutations but not those without the mutations [17].

Early successes with sorafenib were observed in patients with relapsed/refractory FLT3–ITD positive AML before and in those whose disease relapsed following allogeneic stem cell transplant (SCT) [18]. In this report, six patients received sorafenib on compassionate use. The initial dose was 400 mg twice a day and the dose was adjusted in case of cytopenia, suspected toxicity, or resistance. All three patients whose disease relapsed following allogeneic SCT attained CR. Another three patients who had refractory AML achieved CR, facilitating allogeneic SCT in two of the three patients. Since then, two phase I studies have been carried out using sorafenib as maintenance therapy following allogeneic SCT for AML with the FLT3–ITD mutation [19, 20]. Sorafenib was found to be well-tolerated and produced very favorable progression-free survival at 1 year.

Sorafenib has also been studied in combination with chemotherapy for AML patients. When sorafenib (400 mg twice a day) was used in a phase II study with azacytidine (75 mg/m^2^/day × 7 days) in 43 patients with relapsed/refractory AML (40 with FLT3–ITD mutations) [21], an overall response rate (ORR) of 46% was observed. In a phase I/II study of idarubicin (12 mg/m^2^/day × 3) and cytarabine (1.5 g/m^2^/day × 4) induction chemotherapy with sorafenib (400 mg twice a day) as frontline therapy for younger AML patients [22] the CR rate was 75%. With a median follow-up of 54 weeks, the probability of survival at 1 year was 74%. Three subsequent studies have also involved a combination of patients with and without FLT3 mutations. A phase II randomized placebo-controlled study of sorafenib (400 mg twice a day) with daunorubicin (60 mg/m^2^/day × 3) and cytarabine (100 mg/m^2^/day × 7) found that, although CR rates were comparable (60% vs 59%) and adverse events were higher in those who received sorafenib, the median event-free survival (EFS) was significantly longer in the sorafenib arm (21 months vs 9 months) [23]. However, such survival benefit was not observed when a similar regimen was used in elderly AML patients [24], or when sorafenib was used in combination with low-dose cytarabine [25].

The clinical results described above suggest that sorafenib might be effective in reducing leukemic burden and improving progression-free survival (PFS) for patients with relapsed/refractory AML with FLT3–ITD mutations, and may also have a role in combination with chemotherapy in certain patient populations. Further investigation is needed to define the role of sorafenib as frontline therapy, in combination with chemotherapy, for AML with FLT3 mutations, although with the recent Food and Drug Administration (FDA) approval of midostaurin, there may not be as much interest in investigating sorafenib. Since sorafenib is a multi-kinase inhibitor, its role in AML without FLT3 mutations would also be of great interest.

##### Midostaurin

Midostaurin is another oral multi-kinase inhibitor, with activity against not only FLT3 kinase, but also KIT, VEGFR, PDGFR, and protein kinase C. It is currently the only FLT3 inhibitor that is approved by the FDA for use in AML. When used with azacytidine in a phase I/II study for patients with relapsed/refractory AML [26], an ORR of 26% was obtained. The ORR was 33% in those with FLT3–ITD mutations.

In a phase IIb study of midostaurin monotherapy for relapsed/refractory AML assigning patients to either 50 or 100 mg twice a day, an ORR of 71% in the 35 patients with FLT3–ITD mutation and 42% in those without the mutation [27]. A higher midostaurin dose did not improve the outcome. Grade 3/4 non-hematological toxicities included infections, reduction in the ventricular ejection fraction, and diarrhea or nausea/vomiting. A phase Ib study combined midostaurin with daunorubicin (60 mg/m^2^/day × 3) and cytarabine (200 mg/m^2^/day × 7) induction therapy for younger patients with newly diagnosed AML [28]. The initial dose of midostaurin in this study was 100 mg twice a day but the dose had to be reduced to 50 mg twice a day due to toxicities. The combination produced a high CR rate and overall survival (OS). Based on this phase Ib study, a large phase III randomized placebo-controlled RATIFY trial was carried out. In this phase III study, midostaurin (50 mg twice a day) was used in combination with the “3 + 7” regimen as upfront therapy for young AML patients with FLT3 mutations (either ITD or TKD) [29]. Although the CR rates were comparable, patients in the midostaurin arm demonstrated a longer median disease-free survival (DFS) (26.7 months vs 15.5 months) and OS (74.7 months vs 25.6 months). The improved survival benefits were observed even in the patients who subsequently underwent allogeneic SCT, without increased adverse reactions.

The clinical results described above suggest that the addition of midostaurin to the standard “3 + 7” induction regimen as first line therapy might be beneficial for younger AML patients with FLT3 mutations. Since midostaurin is a multi-kinase inhibitor, it would also be interesting to determine its role in combination of chemotherapy for AML without FLT3 mutations.

##### Quizartinib

Quizartinib is an oral kinase inhibitor that is highly selective for FLT3. In a phase I dose escalation study (from 12 to 450 mg/day) in 76 patients with relapsed/refractory AML [30], quizartinib produced an ORR of 17%, but 53% in those with FLT3–ITD mutations. The most common drug-related adverse events were nausea, prolonged QT interval, vomiting, and dysgeusia, most were Grade 2 or lower. Subsequent phase II studies of quizartinib monotherapy in similar groups of patients with FLT3–ITD mutations [31, 32] yielded CR rates of 44–54% and ORRs of 61–72%. These results are extremely compelling, although the duration of remissions in all the cases were short, with the median remission of only 3 months, suggesting the frequent development of resistance to quizartinib. Up to 22% of patients treated with FLT3 inhibitors developed a TKD mutation during FLT3 inhibitor therapy [33].

Quizartinib has also been used in combination with azacytidine or low dose cytarabine in a phase I/II study for relapsed/refractory AML [34]. Among the patients with FLT3–ITD mutations, ORR was high at 73%. Quizartinib has also been used in AML patients with FLT3–ITD mutations whose disease relapsed following allogeneic SCT [35]. The median survival was much improved, compared to historical controls.

Future studies may involve comparing quizartinib with midostaurin to determine if the outcome benefits of midostaurin could be attained with less side effects using a more selective FLT3 inhibitor like quizartinib.

##### Gilteritinib

Gilteritinib is a potent FLT3/AXL inhibitor that shows activities against both FLT3–ITD and FLT3–TKD mutants. In the large phase I/II dose-escalation, dose-expansion Chrysalis trial of gilteritinib monotherapy for relapsed/refractory AML [36], 252 patients, 77% of them with confirmed FLT3 mutations, were assigned to one of seven dose-escalation (20–450 mg/day) cohorts or dose-expansion cohorts. The ORR was 49% in those with FLT3 mutations, but only 12% in those without the mutations. The ORR was higher, at 52%, in those who received ≥80 mg/day of the inhibitor. In this group of patients, the median OS was 31 weeks and the median duration of response was 20 weeks. Gilteritinib was generally well tolerated, with diarrhea and fatigue being the most common adverse reactions.

Preclinical data of gilteritinib combined with azacytidine in AML cells harboring FLT3–ITD mutations showed that the kinase inhibitor augment the apoptosis induced by azacytidine [37], providing the rationale for testing of this combination in the clinic.

##### Crenolanib

Crenolanib is a selective FLT3 inhibitor that is active against both ITD and TKD mutations. It is also uniquely active against leukemic clones that have developed quizartinib resistance [38]. In an open-label phase II study in relapsed/refractory AML with a FLT3 mutation [39]. The ORR was 62% in patients who were FLT3 inhibitor-naïve and 38% in those with a prior history of FLT3 inhibitor therapy. Gastrointestinal toxicity and transaminitis were the most common adverse reactions observed in this study.

Crenolanib has also been used in combination with standard chemotherapy. In a phase II study of idarubicin (12 mg/m^2^/day × 3) and high dose cytarabine (1.5 g/m^2^/day × 4) plus escalating doses of crenolanib (60–100 mg three times a day) in relapsed/refractory AML patients with FLT3 mutations [40], four of the six patients who had failed ≤2 lines of therapy attained CR of their disease. In contrast, none of the five patients who had failed three or more lines of therapy achieved CR.

When crenolanib (100 mg three times a day) was used in combination with the “3 + 7” regimen for newly diagnosed AML patients with FLT3 mutations [41], the overall CR rate was 96%. With a median follow up of 6.2 months, disease relapse was observed in only three of 24 patients.

##### Ongoing clinical trials utilizing FLT3 inhibitors in AML

Based on the availability of an increasing number of FLT3 inhibitors and the encouraging early clinical results obtained using these small molecules, there are currently many clinical trials ongoing internationally to determine the precise role of these inhibitors in the management of AML. A selection of these clinical trials is summarized in Table 1.

**Table 1:** A selection of active studies evaluating FLT3 inhibitors in AML

Protocol title	NCT identifier
Agent: sorafenib	
Phase I/II study of combination of sorafenib, vorinostat, and bortezomib for the treatment of acute myeloid leukemia with complex- or poor-risk (monosomy 5/7) cytogenetics or FLT3–ITD positive genotype (Sponsors: Millennium Pharmaceuticals, Inc., Bayer, and Merck Sharp & Dohme Corp.)	NCT01534260
A pilot study of sorafenib in patients with acute myeloid leukemia as peri-transplant remission maintenance (Sponsor: National Cancer Institute)	NCT01578109
Phase I study of the combination of bortezomib and sorafenib followed by decitabine in patients with acute myeloid leukemia (Sponsor: National Cancer Institute)	NCT01861314
Agent: midostaurin	
An open-labeled, multi-center, expanded treatment protocol (ETP) of midostaurin (PKC412) in patients 18 years of age or older with newly-diagnosed FLT3-mutated acute myeloid leukemia (AML) who are eligible for standard induction and consolidation chemotherapy (Sponsor: Novartis Pharmaceuticals)	NCT03114228
A phase II, randomized trial of standard of care, with or without midostaurin to prevent relapse following allogeneic hematopoietic stem cell transplantation in patients with FLT3–ITD mutated acute myeloid leukemia (Sponsor: Novartis Pharmaceuticals)	NCT01883362
A randomized phase II/III trial of “Novel Therapeutics” versus azacitidine in newly diagnosed patients with acute myeloid leukemia (AML) or high-risk myelodysplastic syndrome (MDS), age 60 or older LEAP: Intergroup less-intense AML platform trial (Sponsor: National Cancer Institute)	NCT03092674
Agent: quizartinib	
A phase 3 open-label randomized study of quizartinib (AC220) monotherapy versus salvage chemotherapy in subjects with tyrosine kinase 3—internal tandem duplication (FLT3–ITD) positive acute myeloid leukemia (AML) refractory to or relapsed after first-line treatment with or without hematopoietic stem cell transplantation (HSCT) consolidation (Sponsor: Daiichi Sankyo Inc.)	NCT02039726
A phase 3, double-blind, placebo-controlled study of quizartinib (AC220) administered in combination with induction and consolidation chemotherapy, and administered as maintenance therapy in subjects 18–75 years old with newly diagnosed FLT3–ITD (+) acute myeloid leukemia (Sponsor: Daiichi Sankyo Inc.)	NCT02668653
A phase II single-arm open-labeled study evaluating combination of quizartinib and omacetaxine mepesuccinate (QUIZOM) in newly diagnosed or relapsed/refractory AML carrying FLT3–ITD (Sponsor: The University of Hong Kong)	NCT03135054
Agent: gilteritinib	
A multi-center, randomized, double-blind, placebo-controlled phase III trial of the FLT3 inhibitor gilteritinib administered as maintenance therapy following allogeneic transplant for patients with FLT3/ITD AML (Sponsor: Astellas Pharma Global Development, Inc.)	NCT02997202
A phase 3 multicenter, randomized, double-blind, placebo-controlled trial of the FLT3 inhibitor gilteritinib (ASP2215) administered as maintenance therapy following induction/consolidation therapy for subjects with FLT3/ITD AML in first complete remission (Sponsor: Astellas Pharma Global Development, Inc.)	NCT02927262
A phase 2/3 multicenter, open-label, 3-arm, 2-stage randomized study of ASP2215 (gilteritinib), combination of ASP2215 plus azacitidine and azacitidine alone in the treatment of newly diagnosed acute myeloid leukemia with FLT3 mutation in patients not eligible for intensive induction chemotherapy (Sponsor: Astellas Pharma Global Development, Inc.)	NCT02752035
Agent: crenolanib	
Pilot study of crenolanib combined with standard salvage chemotherapy in subjects with relapsed/refractory acute myeloid leukemia (Sponsor: Arog Pharmaceuticals, Inc.)	NCT02626338
A phase II study of crenolanib besylate maintenance following allogeneic stem cell transplantation in patients with FLT3-positive acute myeloid leukemia (Sponsor: Arog Pharmaceuticals, Inc.)	NCT02400255
Phase I–II study of crenolanib combined with standard salvage chemotherapy, and crenolanib combined With 5-azacitidine in acute myeloid leukemia patients with FLT3 activating mutations (Sponsor: Arog Pharmaceuticals, Inc.)	NCT02400281
Agent: others	
First in man study to evaluate the safety, tolerability and preliminary efficacy of the Fc-optimized FLT3 antibody FLYSYN for the treatment of acute myeloid leukemia patients with minimal residual disease (Sponsor: University Hospital Tuebingen)	NCT02789254
Phase I open-label, sequential dose escalation study investigating the safety, tolerability, pharmacokinetics, and pharmacodynamics of SKLB1028 when administered daily to patients with relapsed or refractory acute myeloid leukemia (Sponsor: CSPC ZhongQi Pharmaceutical Technology Co., Ltd)	NCT02859948
A first-in-human phase 1/2a study to assess the safety, tolerability, efficacy, and pharmacokinetics of FF-10101-01 in subjects with relapsed or refractory acute myeloid leukemia (Sponsor: Fujifilm Pharmaceuticals U.S.A., Inc.)	NCT03194685

### Targeting epigenetics

#### Epigenetics in AML

Epigenetics refers to the study of mechanisms underlying stable and ideally heritable changes in gene expression or cellular phenotype without changes in the underlying DNA sequences. Various laboratory studies have implicated dysregulation of epigenetic mechanisms in the pathogenesis of AML. In addition, many of the mutations that occur in AML are localized to genes involved in transcriptional regulation [42]. Changes in the genome-wide pattern of methylation are also known to be epigenetic modifiers [43]. Depending on the specific type and site of methylation, the effects on gene expression can differ significantly.

#### Epigenetic mechanisms

**Fig. 2:**
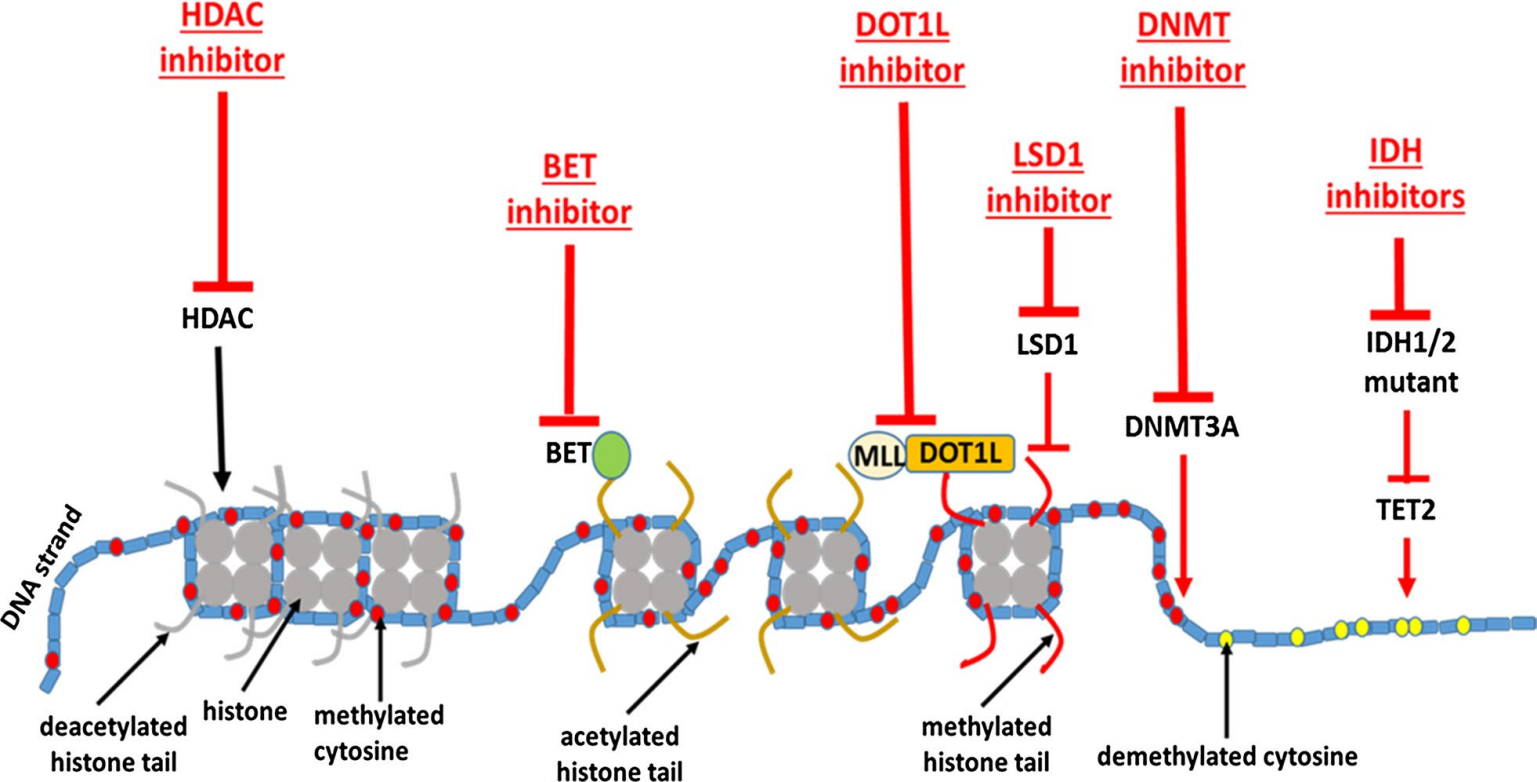
Epigenetic mechanisms of gene regulation through histone acetylation and histone and DNA methylation by various epigenetic modifiers. These epigenetic mechanisms can be blocked by inhibitors at various specified sites

Transcriptional regulation is accomplished through a network of molecular mechanisms (Fig. 2). These include histone acetylation, histone methylation, DNA methylation, and DNA hydroxymethylation. Here, we will limit our discussion to the mechanisms relevant to epigenetic targeting using the currently available small molecules.

##### Histone acetylation and methylation

The first step of gene transcription involves acetylation of the histone tails, causing a change in the chromatin conformation so that the distance between DNA and histone is increased, rendering the DNA more accessible to transcription factors. In contrast, deacetylation induces the opposite effects. Acetylation is catalyzed by histone lysine acetyltransferases (KATs) and deacetylation by HDACs. The acetylated lysine residues are then recognized by bromodomain-containing reader proteins such as the BET proteins [44]. BET proteins include BRD2, BRD3, BRD4, and BRDt.

Transcriptional activation is further modified by histone lysine methylation. Histone lysine methylation is mediated by lysine methyltransferases (KMTs). Histone methylation modulates the affinity of the reader proteins to the histone. Unlike acetylation, histone methylation either activates or represses gene transcription. Molecular abnormalities of the mixed-lineage leukemia (MLL) protein occur recurrently in AML [45]. MLL potentially has more than 70 fusion partners. The MLL protein upregulates Hox expression and results in a block of hematopoietic differentiation [46]. The abnormal fusion proteins arise due to gene translocation or duplication. The abnormal MLL arising from translocation also frequently contains the DOT1L protein [47], a KMT targeting H3K79.

Histone methylation is further modulated by lysine demethylases (KDMs). Lysine-specific histone demethylase 1A (LSD1) is one of the KDMs and has specificity for H3K4 and H3K9. It can function either as a transcriptional activator or repressor.

##### DNA methylation and hydroxymethylation

DNA methylation is catalyzed by DNMTs and converts cytosine residues to 5-methylcytosine. This reaction usually occurs at the CpG islands within the gene and/or at its distant enhancer. DNA methylation usually results in silencing of the specific gene. Mutations in DNMT3A gene occur in more than 20% of AML patients [48]. The frequency increases with age and is associated with poorer clinical outcome.

DNA hydroxymethylation occurs as an intermediary step in the demethylation pathway, oxidizing 5-methylcytosine to 5-hydroxymethylcytosine. This process is catalyzed by TET2 which is mutated in up to 20% of AML cases [43]. DNA hydroxymethylation is dependent on α-ketoglutarate; its conversion from isocitrate is mediated by IDH1 and IDH2. Mutations of IDH1 and IDH2 result in the production of 2-hydroxyglutarate that competitively inhibit TET2 activity [49].

#### Epigenetic modifiers for AML

Based on the major role epigenetics plays in the disease process, targeting epigenetic modifiers represents an attractive option for treating AML (Fig. 2).

##### Histone deacetylase (HDAC) inhibitors

Since HDAC expression is often dysregulated in AML cells [42], targeting HDAC using specific inhibitors has been attempted. However, the clinical response of AML to HDAC inhibitor monotherapy has so far been uniformly disappointing [50, 51], but when combined with chemotherapy, improved response rates were observed. In a phase I study of vorinostat (400 mg/day) used either sequentially or concurrently with decitabine (20 mg/m^2^/day × 5) [52], 2 of 13 AML patients with relapsed/refractory disease treated concurrently attained complete remission but none of the 15 patients treated on the sequentially protocol responded.

A phase II study randomized 149 patients with AML or myelodysplastic syndrome, most treatment-naïve, to receive either azacytidine (50 mg/m^2^/day × 10) monotherapy or azacytidine with entinostat (4 mg/m^2^/day days 3 and 10) [53]. Unfortunately, the addition of entinostat did not improve the hematologic response rate. In contrast, when pracinostat was combined with azacytidine in a phase II study for older AML patients [54], the combination produced a CR and CR with incomplete hematologic recovery (CRi) rate of 42 and 4% respectively. A phase Ib/II study of azacytidine (75 mg/m^2^/day × 5) combined with escalating doses of panobinostat (10–40 mg/day) in intensive chemotherapy-naïve AML and myelodysplastic syndrome (MDS) patients also produced an ORR of 31% for AML and 50% for MDS [55].

HDAC inhibitors have also been used with intensive combination chemotherapy in newly diagnosed AML. A phase Ib/II study combined panobinostat with intensive induction chemotherapy for older patients with newly diagnosed AML [56]. In this study, patients received the standard idarubicin (8 mg/m^2^/day × 3) and cytarabine (100 mg/m^2^/day × 7) regimen plus panobinostat at escalating doses (10–40 mg/day). Patients who attained CR received a consolidation cycle with the same combination, followed by panobinostat maintenance until progression. CR was observed in 64% of the patients, with a time to relapse of 17 months.

When vorinostat was used in combination with idarubicin and cytarabine as induction therapy for AML patients 65 years or younger [57], the ORR was 85% in the group and 100% in those with FLT3–ITD mutations. However, when the identical regimen was used in a phase III randomized study of idarubicin and cytarabine with or without vorinostat [58], no significant clinical benefits were observed in the vorinostat arm.

Based on these results, it is expected that any role HDAC inhibitor may have in the future development of therapeutics for AML will involve combination with chemotherapy.

##### BET inhibitors

BET proteins are crucial in regulating gene transcription and they do so through epigenetic interactions between bromodomains and acetylated histones during cellular proliferation and differentiation processes. BET inhibition has been shown to repress the transcriptional network driven by c-myc [59]. So far, there has only been one reported study of a BET inhibitor for patients with AML. In a phase I dose-escalation study of monotherapy with the bromodomain OTX015 in adult acute leukemia patients (36 with AML) who had failed or were unable to receive standard induction chemotherapy [60], three patients attained a CR or CRi and two other patients had partial blast clearance. Diarrhea and fatigue were common adverse reactions, and two of the patients developed hyperbilirubinemia.

##### DOT1L inhibitors

The DOT1L inhibitor, pinometostat, has shown activity in animal models of acute leukemia [61]. It also increased the in vitro sensitivity of MLL-rearranged AML to chemotherapy [62]. In a phase I study of pinometostat monotherapy in patients with relapsed/refractory acute leukemia [63], clinical response was observed in six of the 49 patients, with two patients attaining CR, one PR, and three resolution of leukemia cutis. Adverse events included nausea, constipation, vomiting, abdominal pain, diarrhea, hypocalcemia, hypokalemia, hypomagnesemia, fatigue, fever, peripheral edema, mucositis, febrile neutropenia, leukocytosis, anemia, cough, dyspnea, and pneumonia. Interestingly, nine patients showed evidence of differentiation syndrome.

##### LSD1 inhibitors

Leukemia cells, including those with complex cytogenetics have so far been consistently demonstrated in vitro to be highly sensitive to LSD1 inhibitors [64–67]. The LSD1 inhibitor T-3775440 has been shown to disrupt the transcription factor, growth factor-independent 1B (GFI1B) complex and impede leukemia cell growth [65]. The LSD1 inhibitors NCD25 and NCD38 hindered the oncogenic potentials of leukemia cell lines [67]. Although human studies are ongoing, no clinical results are currently available.

##### DNMT inhibitors

Azacytidine and decitabine are two DNMT inhibitors that have been used alone or in combination with low dose cytarabine for treating AML in patients who are not suitable candidates for intensive induction chemotherapy. They both produced CR and CRi rates around 20% [68–71]. Since the DNMT inhibitors azacytidine and decitabine have already been used in the clinic extensively to treat AML and MDS, we will limit our discussion of DNMT inhibitors in the review to the second generation DNMT inhibitor, guadecitabine.

Guadecitabine is also known as SGI-110 and is a novel hypomethylating dinucleotide of decitabine and deoxyguanosine. Unlike azacytidine and decitabine, it is resistant to degradation by cytidine deaminase. A phase I multicenter, dose-escalation randomized study assigned 35 patients with AML and nine patients with myelodysplastic syndrome (MDS) in the daily ×5 dose-escalation cohorts, 28 patients with AML and six patients with MDS in the once-weekly dose-escalation cohorts, and 11 patients with AML and four patients with MDS in the twice-weekly dose-escalation cohorts [72]. Six of the 74 patients with AML and six of the 19 patients with MDS had a clinical response to treatment. The most common Grade 3 or higher adverse events were febrile neutropenia, pneumonia, thrombocytopenia, anemia, and sepsis.

##### IDH inhibitors

IDH1 and IDH2 mutations occur in around 5–10 and 10–15% of adult AML respectively [73]. Interestingly, IDH mutations predict for response to therapeutic BCL-2 inhibition [3]. Several IDH inhibitors are currently being investigated clinically. IDH305 suppresses mutant IDH1-dependent 2-hydroxyglutarate production and was tested as monotherapy in a phase I study that included 21 patients with relapsed/refractory AML [74]. CR was observed in 2, CRi 1, and PR 4 patients. Another phase I study used a different IDH1 inhibitor, AG-120, as monotherapy in 78 patients with mutant IDH1, 63 of these patients had relapsed/refractory AML [75]. ORR was observed in 38% and CR 18%. The median duration of response was 10.2 months for all responders and 6.5 months for the R/R AML responding patients. The majority of adverse events observed in these two studies were Grade 1/2, including diarrhea, fatigue, nausea, fever, and IDH inhibitor-associated differentiation syndrome.

Enasidenib is an IDH2 inhibitor. In a multicenter phase I/II study of 239 patients [76], ORR of 40.3% was observed among the 176 patients evaluable for efficacy when it was given as monotherapy, with a median duration of response of 5.8 months. The median OS among the relapsed/refractory patients was 9.3 months, and for the 34 patients (19.3%) who attained CR was 19.7 months. Grade 3/4 enasidenib-related adverse events included indirect hyperbilirubinemia (12%) and IDH inhibitor-associated differentiation syndrome (7%), which is characterized by fever, edema, hypotension, malaise, and pleural and/or pericardial effusions, in addition to marked neutrophil-predominant leukocytosis.

##### Ongoing clinical trials utilizing epigenetic modifiers in AML

Based on the availability of an increasing number of epigenetic modifiers and the encouraging early clinical results obtained using these small molecules, there are currently many clinical trials ongoing internationally to determine the precise role of these inhibitors in the management of AML. A selection of these clinical trials is summarized in Table 2.

**Table 2:** A selection of active studies evaluating epigenetic modifiers in AML

Protocol title	NCT identifier
Agent: HDAC inhibitors	
A phase 2 study of temozolomide plus vorinostat in patients with relapse/refractory acute myeloid leukemia (AML) (Sponsor: Stanford University)	NCT01550224
Phase I/II study with oral panobinostat maintenance therapy following allogeneic stem cell transplantation in patients with high risk MDS or AML (PANOBEST) (Sponsor: Johann Wolfgang Goethe University Hospital)	NCT01451268
A phase 1 study of AZD1775 in combination with belinostat in relapsed and refractory myeloid malignancies and selected untreated patients with acute myeloid leukemia (Sponsor: National Cancer Institute)	NCT02381548
A phase I and dose expansion cohort study of panobinostat in combination with fludarabine and cytarabine in pediatric patients with refractory or relapsed acute myeloid leukemia or myelodysplastic syndrome (Sponsor: St. Jude Children’s Research Hospital)	NCT02676323
Agent: BET inhibitors	
A phase 1/2, open-label, dose-escalation, safety and tolerability study of INCB054329 in subjects with advanced malignancies (Sponsor: Incyte Corporation)	NCT02431260
A phase I/II open-label, dose escalation study to investigate the safety, pharmacokinetics, pharmacodynamics and clinical activity of GSK525762 in subjects with relapsed, refractory hematologic malignancies (Sponsor: GlaxoSmithKline)	NCT01943851
A phase 1 dose escalation, multicenter, open-label, safety, pharmacokinetic and pharmacodynamic study of FT-1101 in patients with relapsed or refractory hematologic malignancies (Sponsor: Forma Therapeutics, Inc.)	NCT02543879
A dose escalation study of RO6870810/TEN-010 in patients with acute myeloid leukemia and myelodysplastic syndrome (Sponsor: Hoffmann-La Roche)	NCT02308761
A phase 1/2, open-label, dose-escalation, safety and tolerability study of INCB054329 in subjects with advanced malignancies (Sponsor: Incyte Corporation)	NCT02431260
Agent: LSD1 inhibitors	
A phase 1/2, open-label, dose-escalation/dose-expansion, safety and tolerability study of INCB059872 in subjects with advanced malignancies (Sponsor: Incyte Corporation)	NCT02712905
A phase I open-label, dose escalation study to investigate the safety, pharmacokinetics, pharmacodynamics and clinical activity of GSK2879552 given orally in subjects with relapsed/refractory acute myeloid leukemia (Sponsor: GlaxoSmithKline)	NCT02177812
A multi-center, open label study to assess the safety, steady-state pharmacokinetics and pharmacodynamics of IMG-7289 with and without ATRA (Tretinoin) in patients with advanced myeloid malignancies (Sponsor: Imago BioSciences, Inc.)	NCT02842827
Agent: guadecitabine	
A phase 3, multicenter, randomized, open-label study of guadecitabine (SGI-110) versus treatment choice in adults with previously treated acute myeloid leukemia (Sponsor: Astex Pharmaceuticals)	NCT02920008
A phase 3, multicenter, open-label, randomized study of SGI-110 versus treatment choice (TC) in adults with previously untreated acute myeloid leukemia (AML) who are not considered candidates for intensive remission induction chemotherapy (Sponsor: Astex Pharmaceuticals)	NCT02348489
A phase Ib study evaluating the safety and pharmacology of atezolizumab (Anti-PD-L1 Antibody) administered in combination with immunomodulatory agents in patients with acute myeloid leukemia (Sponsor: Hoffmann-La Roche)	NCT02892318
Agent: IDH inhibitors	
An open-label, non-randomized, multicenter phase I study to determine the maximum tolerated and/or recommended phase II dose of oral mutant IDH1 (mIDH1) inhibitor BAY1436032 and to characterize its safety, tolerability, pharmacokinetics, pharmacodynamics, and preliminary clinical efficacy in patients with mIDH1-R132X advanced acute myeloid leukemia (AML) (Sponsor: Bayer)	NCT03127735
A phase 1, multicenter, open-label, safety study of AG-120 or AG-221 in combination with induction therapy and consolidation therapy in patients with newly diagnosed acute myeloid leukemia with an IDH1 and/or IDH2 mutation (Sponsor: Agios Pharmaceuticals, Inc.)	NCT02632708
A phase 3, multicenter, double-blind, randomized, placebo-controlled study of AG-120 in combination with azacitidine in subjects ≥18 years of age with previously untreated acute myeloid leukemia with an IDH1 mutation (Sponsor: Agios Pharmaceuticals, Inc.)	NCT03173248
A phase 1/1b, multicenter, open-label, dose-escalation study of FT-2102 as a single agent and in combination with azacitidine in patients with acute myeloid leukemia or myelodysplastic syndrome with an IDH1 mutation (Sponsor: Forma Therapeutics, Inc.)	NCT02719574

### Targeting BCL-2 and JAK/STAT pathway

**Fig. 3:**
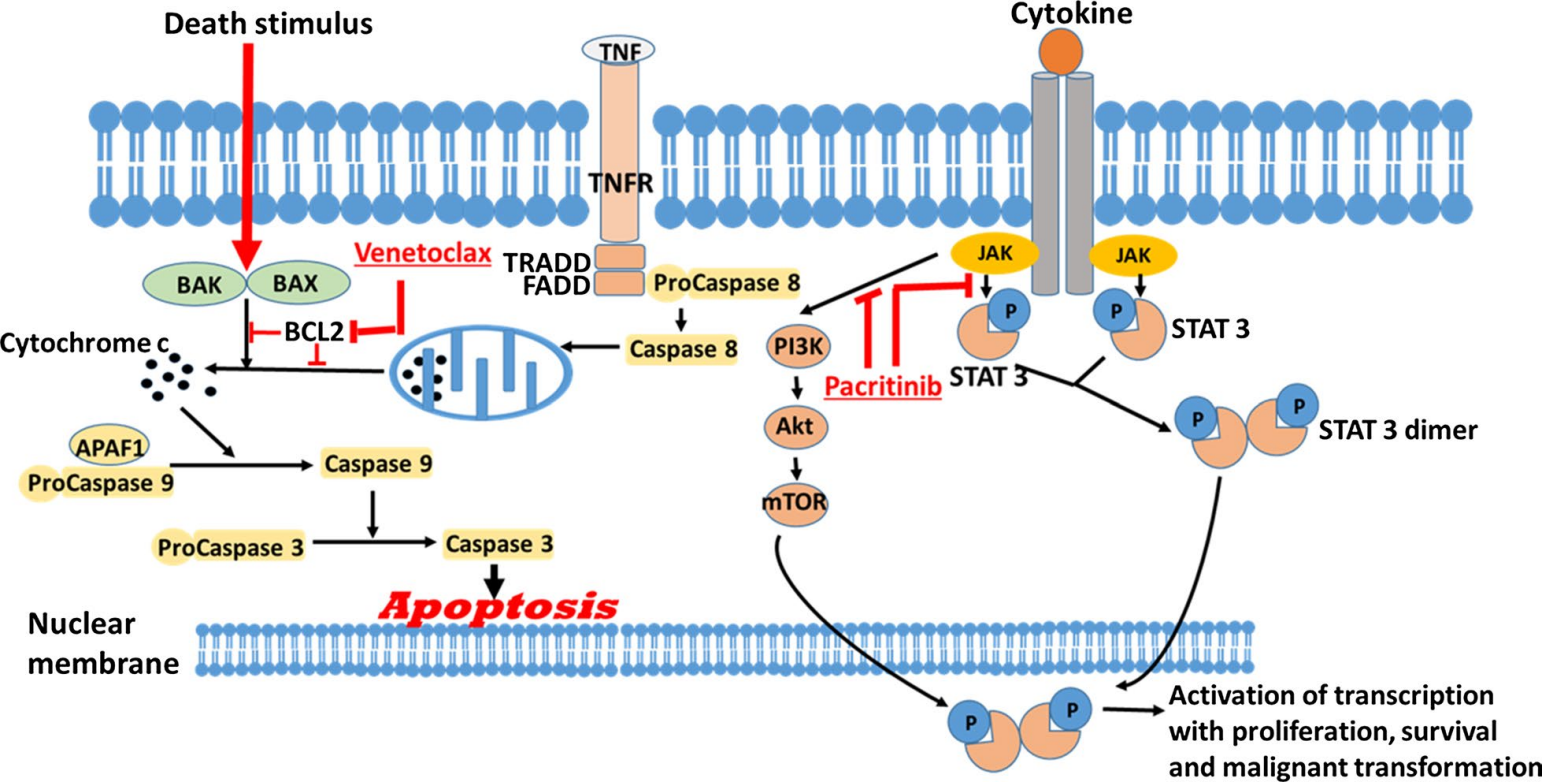
BCL-2 and JAK/STAT pathways showing how BCL-2 inhibitors affect leukemia cell apoptosis and JAK/STAT inhibitors affect the proliferation of leukemia cells

BCL-2 is an anti-apoptotic protein that has been demonstrated to induce chemoresistance, and overexpression has been implicated in AML [77] (Fig. 3). Venetoclax is an oral BCL-2 inhibitor currently being investigated for AML. It appears to be particularly effective in patients with IDH1/2 mutations [3]. When used as monotherapy in a phase II study of patients with relapsed/refractory AML [78], 19% overall response rate was observed, with another 19% showing antileukemic activity not meeting the IWG criteria for response. Three of the twelve patients with IDH1/2 mutations achieved CR or CRi. Common adverse events included nausea, diarrhea and vomiting, and febrile neutropenia and hypokalemia (Grade 3/4). Hox expression also predicts response to venetoclax [79].

In two phase I studies of venetoclax combined with low dose chemotherapy for chemotherapy-naïve AML patients aged 65 years or older, high response rates were obtained. In the study that combined venetoclax with either azacytidine or decitabine [80], responses were obtained in 26 (76%) of the 34 evaluable patients, with 13 CR and 11 CRi. Eleven patients had IDH1/2 mutations, of whom nine (82%) responded. In a trial that combined venetoclax with low dose cytarabine [81], a 44% ORR was observed in the 18 patients treated, with four patients attaining CR and another four CRi.

Targeting the JAK/STAT pathway is another molecular therapeutic option since JAK mutation has been implicated in some patients with AML [82]. In a phase I/II study of pacritinib, a JAK/STAT inhibitor, in patients with advanced myeloid malignancies [83], three of the seven patients treated for AML were reported to show clinical benefits. Pacritinib was well tolerated and the most frequent adverse reactions were diarrhea, nausea, vomiting, and fatigue, most were Grade 1/2, with Grade 3 side effects reported in 22.6%, four of whom had diarrhea. Being an inhibitor of the JAK/STAT pathway, pacritinib may also be effective in AML with FLT3 mutations. Further investigations are, therefore, merited.

#### Ongoing clinical trials targeting BCL-2 and JAK/STAT pathway in AML

There are currently many clinical trials ongoing internationally to determine the precise role of these inhibitors in the management of AML. A selection of these clinical trials is summarized in Table 3.

**Table 3:** A selection of active studies evaluating BCL-2 and JAK/STAT inhibitors in AML

Protocol title	NCT identifier
Agent: BCL-2 inhibitors	
Phase I dose-escalation study of the orally administered selective Bcl-2 Inhibitor S 055746 as monotherapy for the treatment of patients with acute myeloid leukaemia (AML) or high or very high risk myelodysplastic syndrome (MDS) (Sponsor: Institut de Recherches Internationales Servier)	NCT02920541
A phase 2a, open-label, dose-escalation study evaluating the safety, pharmacokinetics, pharmacodynamics, and clinical effects of intravenously administered nerofe in subjects with acute myelogenous leukemia or myelodysplastic syndrome (Sponsor: Immune System Key Ltd)	NCT03059615
A phase 1b study evaluating the safety, pharmacokinetics and efficacy of venetoclax as a single-agent and in combination with azacitidine in subjects with higher-risk myelodysplastic syndromes after hypomethylating agent-failure (Sponsor: AbbVie)	NCT02966782
A phase I and expansion cohort study of venetoclax in combination with chemotherapy in pediatric patients with refractory or relapsed acute myeloid leukemia (Sponsor: St. Jude Children’s Research Hospital)	NCT03194932
A randomized, double-blind, placebo controlled study of venetoclax co-administered with low dose cytarabine versus low dose cytarabine in treatment naïve patients with acute myeloid leukemia who are ineligible for intensive chemotherapy (Sponsor: AbbVie)	NCT03069352
A randomized, double-blind, placebo controlled phase 3 study of venetoclax in combination with azacitidine versus azacitidine in treatment naïve subjects with acute myeloid leukemia who are ineligible for standard induction therapy (Sponsor: AbbVie)	NCT02993523
A phase 1b study of ABT-199 (GDC-0199) in combination with azacitidine or decitabine in treatment-naive subjects with acute myelogenous leukemia who are ≥60 years of age and who are not eligible for standard induction therapy (Sponsor: AbbVie)	NCT02203773
Agent: JAK/STAT inhibitors	
Induction therapy with pacritinib combined with decitabine or cytarabine in older patients with acute myeloid leukemia (AML) (Sponsor: Weill Medical College of Cornell University)	NCT02532010
Phase I study of pacritinib and chemotherapy in patients with acute myeloid leukemia and FLT3 mutations (Sponsor: Ohio State University Comprehensive Cancer Center)	NCT02323607

## Conclusions

The lack of improvement in the outcome of AML with standard chemotherapeutic agents suggests the need to explore other therapeutic approaches. Molecular targeting holds great promise. The molecular mechanisms most intensely targeted are the FLT3 signaling pathway, epigenetics, and the BCL-2 and JAK/STAT pathways. The inhibitors discussed in this review have all shown significant activity against AML. However, there remain many questions to be answered before these agents can provide the next leap in the prognosis of AML patients. These questions include what the exact role these compounds should be in clinical practice, whether they should be used in combination with chemotherapy, the timing of the targeted therapy, and the role of maintenance therapy following either consolidation therapy or allogeneic SCT. Furthermore, the multiple co-active intracellular pathways and the genomic instability in AML provide the opportunity for the AML cells to develop additional mutations, rendering them resistant to the inhibitors. The heterogeneity of the disease among AML patients is also another potential obstacle. With advances in the understanding of the molecular events associated with AML and the inclusion of genome-wide sequencing in the routine investigations in AML patients, it is likely that personalized medicine will herald a new era in AML therapy. This is especially so with the increasing number of compound being made available. Not only will these compounds be routinely combined with conventional chemotherapy during induction or consolidation therapy, sequential application of different molecular inhibitors may also be used in individual AML patients, according to changes in the genomic landscape of the leukemia cells. AML therapy will no longer be “one size fits all”.

## Abbreviations

AML: acute myeloid leukemia
FLT3: FMS-like tyrosine kinase 3
DNMT: DNA methyltransferase
TET: ten-eleven-translocation
IDH: isocitrate dehydrogenase
HDAC: histone deacetylase
BET: bromodomain and extra terminal
DOT1L: disruptor of telomeric silencing 1-like
BCL-2: B cell lymphoma-2
PML-RARα: promyelocytic leukemia-retinoic acid receptor alpha
APL: acute promyelocytic leukemia
JAK: Janus kinase
ITD: internal tandem duplication
TKD: tyrosine kinase domain
CR: complete remission
ROS: reactive oxygen species
VEGFR: vascular endothelial growth factor receptor
PDGFR: platelet-derived growth factor receptor
SCT: stem cell transplant
ORR: overall response rate
EFS: event-free survival
PFS: progression-free survival
FDA: Food and Drug Administration
OS: overall survival
DFS: disease-free survival
KAT: histone lysine acetyltransferase
KMT: lysine methyltransferase
KDM: lysine demethylase
MLL: mixed-lineage leukemia
LSD1: lysine-specific histone demethylase
MDS: myelodysplastic syndrome
CRi: complete remission with incomplete blood count recovery

## References

[1] Hann IM, Stevens RF, Goldstone AH, Rees JKH, Wheatley K, Gray RG (1997). Randomized comparison of DAT versus ADE as induction chemotherapy in children and younger adults with acute myeloid leukemia. Results of the Medical Research Council’s 10th AML Trial (MRC AML10). Blood.

[2] Grimwade D, Hills RK, Moorman AV, Walker H, Chatters S, Goldstone AH (2010). Refinement of cytogenetic classification in acute myeloid leukemia: determination of prognostic significance of rare recurring chromosomal abnormalities among 5876 younger adult patients treated in the United Kingdom Medical Research Council trials. Blood.

[3] Chan SM, Thomas D, Corces-Zimmerman MR, Xavy S, Rastogi S, Hong WJ (2015). Isocitrate dehydrogenase 1 and 2 mutations induce BCL-2 dependence in acute myeloid leukemia. Nat Med.

[4] Takahashi S (2006). Inhibition of the MEK/MAPK signal transduction pathway strongly impairs the growth of Flt3-ITD cells. Am J Hematol.

[5] Hayakawa F, Towatari M, Kiyoi H, Tanimoto M, Kitamura T, Saito H (2000). Tandem-duplicated Flt3 constitutively activates STAT5 and MAP kinase and introduces autonomous cell growth in IL-3-dependent cell lines. Oncogene.

[6] Fu L, Fu H, Wu Q, Pang Y, Xu K, Zhou L (2017). High expression of ETS2 predicts poor prognosis in acute myeloid leukemia and may guide treatment decisions. J Transl Med.

[7] Levis M, Small D (2003). FLT3: ITDoes matter in leukemia. Leukemia.

[8] Gilliland DG, Griffin JD (2002). The roles of FLT3 in hematopoiesis and leukemia. Blood.

[9] Yamamoto Y, Kiyoi H, Nakano Y, Suzuki R, Kodera Y, Miyawaki S (2001). Activating mutation of D835 within the activation loop of FLT3 in human hematologic malignancies. Blood.

[10] Zheng R, Levis M, Piloto O, Brown P, Baldwin BR, Gorin NC (2004). FLT3 ligand causes autocrine signaling in acute myeloid leukemia cells. Blood.

[11] Ozeki K, Kiyoi H, Hirose Y, Iwai M, Ninomiya M, Kodera Y (2004). Biologic and clinical significance of the FLT3 transcript level in acute myeloid leukemia. Blood.

[12] Choudhary C, Schwable J, Brandts C, Tickenbrock L, Sargin B, Kindler T (2005). AML-associated Flt3 kinase domain mutations show signal transduction differences compared with Flt3 ITD mutations. Blood.

[13] Mizuki M, Schwable J, Steur C, Choudhary C, Agrawal S, Sargin B (2003). Suppression of myeloid transcription factors and induction of STAT response genes by AML-specific Flt3 mutations. Blood.

[14] Grundler R, Miething C, Thiede C, Peschel C, Duyster J (2005). FLT3-ITD and tyrosine kinase domain mutants induce 2 distinct phenotypes in a murine bone marrow transplantation model. Blood.

[15] Kim KT, Baird K, Ahn JY, Meltzer P, Lilly M, Levis M (2005). Pim-1 is upregulated by constitutively activated FLT3 and plays a role in FLT3-mediated cell survival. Blood.

[16] Sallmyr A, Fan J, Datta K, Kim KT, Grosu D, Shapiro P (2008). Internal tandem duplication of FLT3 (FLT3/ITD) induces increased ROS production, DNA damage, and misrepair: implications for poor prognosis in AML. Blood.

[17] Zhang W, Konopleva M, Shi YX, McQueen T, Harris D, Ling X (2008). Mutant FLT3: a direct target of sorafenib in acute myelogenous leukemia. J Natl Cancer Inst.

[18] Metzelder S, Wang Y, Wollmer E, Wanzel M, Teichler S, Chaturvedi A (2009). Compassionate use of sorafenib in FLT3-ITD—positive acute myeloid leukemia: sustained regression before and after allogeneic stem cell transplantation. Blood.

[19] Chen Y-B, Shuli L, Andrew LA, Connolly C, Del Rio C, Valles B (2014). Phase I trial of maintenance sorafenib after allogeneic hematopoietic stem cell transplantation for patients with FLT3-ITD AML. Blood.

[20] Battipaglia G, Ruggeri A, Massoud R, El Cheikh J, Jestin M, Antar A (2017). Efficacy and feasibility of sorafenib as a maintenance agent after allogeneic hematopoietic stem cell transplantation for Fms-like tyrosine kinase 3-mutated acute myeloid leukemia. Cancer.

[21] Ravandi F, Alattar ML, Grunwald MR, Rudek MA, Rajkhowa T, Richie MA (2013). Phase 2 study of azacytidine plus sorafenib in patients with acute myeloid leukemia and FLT-3 internal tandem duplication mutation. Blood.

[22] Ravandi F, Cortes JE, Jones D, Faderl S, Garcia-Manero G, Konopleva MY (2010). Phase I/II study of combination therapy with sorafenib, idarubicin, and cytarabine in younger patients with acute myeloid leukemia. J Clin Oncol.

[23] Röllig C, Serve H, Hüttmann A, Noppeney R, Müller-Tidow C, Krug U, Baldus CD (2015). Addition of sorafenib versus placebo to standard therapy in patients aged 60 years or younger with newly diagnosed acute myeloid leukaemia (SORAML): a multicentre, phase 2, randomised controlled trial. Lancet Oncol.

[24] Serve H, Krug U, Wagner R, Sauerland MC, Heinecke A, Brunnberg U (2013). Sorafenib in combination with intensive chemotherapy in elderly patients with acute myeloid leukemia: results from a randomized, placebo-controlled trial. J Clin Oncol.

[25] MacDonald DA, Assouline AS, Brandwein J, Kamel-Reid S, Eisenhauer EA, Couban S (2013). A phase I/II study of sorafenib in combination with low dose cytarabine in elderly patients with acute myeloid leukemia or high-risk myelodysplastic syndrome from the National Cancer Institute of Canada Clinical Trials Group: trial IND.186.. Leuk Lymphoma.

[26] Strati P, Kantarjian H, Ravandi F, Nazha A, Borthakur G, Daver N (2015). Phase I/II trial of the combination of midostaurin (PKC412) and 5-azacytidine for patients with acute myeloid leukemia and myelodysplastic syndrome. Am J Hematol.

[27] Fischer T, Stone RM, Deangelo DJ, Galinsky I, Estey E, Lanza C (2010). Phase IIB trial of oral midostaurin, the FMS-like tyrosine kinase receptor and multi-targeted inhibitor in patients with acute myeloid leukemia and high-risk myelodysplastic syndrome with either wildtype or mutated FLT3. J Clin Oncol.

[28] Stone RM, Fischer T, Paquette R, Schiller G, Schiffer CA, Ehninger G (2012). Phase IB study of the FLT3 kinase inhibitor midostaurin with chemotherapy in younger newly diagnosed adult patients with acute myeloid leukemia. Leukemia.

[29] Stone RM, Mandreka SJ, Sanford BL, Laumann K, Geyer S, Bloomfield CD (2017). Midostaurin plus chemotherapy for acute myeloid leukemia with a FLT3 mutation. N Engl J Med.

[30] Cortes JE, Kantarjian H, Foran JM, Ghirdaladze D, Zodelava M, Borthakur G (2013). Phase I study of quizartinib administered daily to patients with relapsed or refractory acute myeloid leukemia irrespective of FMS-like tyrosine kinase 3-internal tandem duplication status. J Clin Oncol.

[31] Levis MJ, Perl A, Dombret H, Döhner M, Steffen B, Rousselot P (2012). Final results of a phase 2 open-label, monotherapy efficacy and safety study of quizartinib (AC220) in patients with FLT3-ITD positive or negative relapsed/refractory acute myeloid leukemia after second-line chemotherapy or hematopoietic stem cell transplantation. Blood.

[32] Cortes JE, Perl A, Dombret H, Kayser S, Steffen B, Rousselot P (2012). Final results of a phase 2 open-label, monotherapy efficacy and safety study of quizartinib (AC220) in patients ≥60 years of age with FLT3 ITD positive or negative relapsed/refractory acute myeloid leukemia. Blood.

[33] Alvarado Y, Kantarjian HM, Luthra R, Ravandi F, Borthakur G, Garcia-Manero G (2014). Treatment with FLT3 inhibitor in patients with FLT3-mutated acute myeloid leukemia is associated with development of secondary FLT3-tyrosine kinase domain mutations. Cancer.

[34] Abdelall WKH, Borthakur G, Garcia-Manero G, Patel KP, Jabbour EJ, Daver NG (2016). The combination of quizartinib with azacitidine or low dose cytarabine is highly active in patients (Pts) with FLT3-ITD mutated myeloid leukemias: interim report of a phase I/II trial. Blood.

[35] Hills RK, Gammon G, Trone D, Burnett AK (2015). Quizartinib significantly improves overall survival in FLT3-ITD positive AML patients relapsed after stem cell transplantation or after failure of salvage chemotherapy: a comparison with historical AML database (UK NCRI data). Blood.

[36] Perl AE, Altman JK, Cortes JE, Smith CC, Litzow M, Baer MR (2016). Final results of the Chrysalis trial: a first-in-human phase 1/2 dose-escalation, dose-expansion study of gilteritinib (ASP2215) in patients with relapsed/refractory acute myeloid leukemia (R/R AML). Blood.

[37] Ueno Y, Mori M, Kamiyama Y, Kaneko N, Isshiki E, Takeuchi M (2016). Gilteritinib (ASP2215), a novel FLT3/AXL inhibitor: preclinical evaluation in combination with azacitidine in acute myeloid leukemia. Blood.

[38] Smith CC, Lasater EA, Lin KC, Wang Q, McCreery MQ, Stewart WK (2014). Crenolanib is a selective type I pan-FLT3 inhibitor. Proc Natl Acad Sci.

[39] Randhawa JK, Kantarjian H, Borthakur G, Thompson PA, Konopleva M, Daver N (2014). Results of a phase II study of crenolanib in relapsed/refractory acute myeloid leukemia patients (Pts) with activating FLT3 mutations. Blood.

[40] Ohanian M, Kantarjian HM, Borthakur G, Kadia TM, Konopleva M, Garcia-Manero G (2016). Efficacy of a type I FLT3 inhibitor, crenolanib, with idarubicin and high-dose ara-C in multiply relapsed/refractory FLT3+ AML. Blood.

[41] Wang ES, Stone RM, Tallman MS, Walter RB, Eckardt JR, Collins R (2016). Crenolanib, a type I FLT3 TKI, can be safely combined with cytarabine and anthracycline induction chemotherapy and results in high response rates in patients with newly diagnosed FLT3 mutant acute myeloid leukemia (AML). Blood.

[42] Abdel-Wahab O, Levine RL (2013). Mutations in epigenetic modifiers in the pathogenesis and therapy of acute myeloid leukemia. Blood.

[43] Cancer Genome Atlas Research Network (2013). Genomic and epigenomic landscapes of adult de novo acute myeloid leukemia. N Engl J Med.

[44] Umehara T, Nakamura Y, Jang MK, Nakano K, Tanaka A, Ozato K (2010). Structural basis for acetylated histone H4 recognition by the human BRD2 bromodomain. J Biol Chem.

[45] Sorensen PH, Chen CS, Smith FO, Arthur DC, Domer PH, Bernstein ID (1994). Molecular rearrangements of the MLL gene are present in most cases of infant acute myeloid leukemia and are strongly correlated with monocytic or myelomonocytic phenotypes. J Clin Investig.

[46] Kawagoe H, Humphries RK, Blair A, Sutherland HJ, Hogge DE (1999). Expression of HOX genes, HOX cofactors, and MLL in phenotypically and functionally defined subpopulations of leukemic and normal human hematopoietic cells. Leukemia.

[47] Okada Y, Feng Q, Lin Y, Jiang Q, Li Y, Coffield VM (2005). hDOT1L links histone methylation to leukemogenesis. Cell.

[48] Ley TJ, Ding L, Walter MJ, McLellan MD, Lamprecht T, Larson DE (2010). DNMT3A mutations in acute myeloid leukemia. N Engl J Med.

[49] Ward PS, Patel J, Wise DR, Abdel-Wahab O, Bennett BD, Coller HA (2010). The common feature of leukemia-associated IDH1 and IDH2 mutations is a neomorphic enzyme activity converting alpha-ketoglutarate to 2-hydroxyglutarate. Cancer Cell.

[50] Garcia-Manero G, Yang H, Bueso-Ramos C, Ferrajoli A, Cortes J, Wierda WG (2008). Phase 1 study of the histone deacetylase inhibitor vorinostat (suberoylanilide hydroxamic acid [SAHA]) in patients with advanced leukemias and myelodysplastic syndromes. Blood.

[51] Gojo I, Jiemjit A, Trepel JB, Sparreboom A, Figg WD, Rollins S (2007). Phase 1 and pharmacologic study of MS-275, a histone deacetylase inhibitor, in adults with refractory and relapsed acute leukemias. Blood.

[52] Kirschbaum M, Gojo I, Goldberg SL, Bredeson C, Kujawski LA, Yang A (2014). A phase 1 clinical trial of vorinostat in combination with decitabine in patients with acute myeloid leukaemia or myelodysplastic syndrome. Br J Haematol.

[53] Prebet T, Sun Z, Figueroa ME, Ketterling R, Melnick A, Greenberg PL (2014). Prolonged administration of azacitidine with or without entinostat for myelodysplastic syndrome and acute myeloid leukemia with myelodysplasia-related changes: results of the US Leukemia Intergroup Trial E1905. J Clin Oncol.

[54] Garcia-Manero G, Othus M, Pagel JM, Radich JP, Fang M, Rizzieri DA (2016). SWOG S1203: a randomized phase III study of standard cytarabine plus daunorubicin (7 + 3) therapy versus idarubicin with high dose cytarabine (IA) with or without vorinostat (IA + V) in younger patients with previously untreated acute myeloid leukemia (AML). Blood.

[55] Tan P, Wei A, Mithraprabhu S, Cummings N, Liu HB, Perugini M (2014). Dual epigenetic targeting with panobinostat and azacitidine in acute myeloid leukemia and high-risk myelodysplastic syndrome. Blood Cancer J.

[56] Ocio EM, Herrera P, Olave M-T, Castro N, Pérez-Simón JA, Brunet S (2015). Panobinostat as part of induction and maintenance for elderly patients with newly diagnosed acute myeloid leukemia: phase Ib/II panobidara study. Haematologica.

[57] Garcia-Manero G, Tamboro FP, Bekele NB, Yang H, Ravandi F, Jabbour E (2012). Phase II trial of vorinostat with idarubicin and cytarabine for patients with newly diagnosed acute myelogenous leukemia or myelodysplastic syndrome. J Clin Oncol.

[58] Garcia Manero G, Atallah E, Khaled SK, Arellano M, Patnaik MM, Odenike O (2016). A phase 2 study of pracinostat and azacitidine in elderly patients with acute myeloid leukemia (AML) not eligible for induction chemotherapy: response and long-term survival benefit. Blood.

[59] Coudé MM, Braun T, Berrou J, Dupont M, Bertrand S, Masse A (2015). BET inhibitor OTX015 targets BRD2 and BRD4 and decreases c-MYC in acute leukemia cells. Oncotarget.

[60] Berthon C, Raffoux E, Thomas X, Vey N, Gomez-Roca C, Yee K (2016). Bromodomain inhibitor OTX015 in patients with acute leukaemia: a dose-escalation, phase 1 study. Lancet Haematol.

[61] Rau RE, Rodriguez B, Luo M, Jeong M, Rosen A, Rogers JH (2016). DOT1L as a therapeutic target for the treatment of DNMT3A-mutant acute myeloid leukemia. Blood.

[62] Liu W, Deng L, Song Y, Redell M (2014). DOT1L inhibition sensitizes MLL-rearranged AML to chemotherapy. PLoS ONE.

[63] Stein EM, Garcia-Manero G, Rizzieri DA, Tibes R, Berdeja JG, Jongen-Lavrencic M (2015). A phase 1 study of the DOT1L inhibitor, pinometostat (EPZ-5676), in adults with relapsed or refractory leukemia: safety, clinical activity, exposure and target inhibition. Blood.

[64] Fiskus W, Sharma S, Shah B, Portier BP, Devaraj SG, Liu K (2017). Highly effective combination of LSD1 (KDM1A) antagonist and pan-histone deacetylase inhibitor against human AML cells. Leukemia.

[65] Ishikawa Y, Gamo K, Yabuki M, Takagi S, Toyoshima K, Nakayama K (2017). A novel LSD1 inhibitor T-3775440 disrupts GFI1B-containing complex leading to transdifferentiation and impaired growth of AML cells. Mol Cancer Ther.

[66] Sugino N, Kawahara M, Tatsumi G, Kanai A, Matsui H, Yamamoto R (2017). A novel LSD1 inhibitor NCD38 ameliorates MDS-related leukemia with complex karyotype by attenuating leukemia programs via activating super-enhancers. Leukemia.

[67] Fiskus W, Sharma S, Shah B, Portier BP, Devaraj SG, Liu K (2017). Highly effective combination of LSD1 (KDM1A) antagonist and pan-histone deacetylase inhibitor against human AML cells. Leukemia.

[68] Pleyer L, Burgstaller S, Girschikofsky M, Linkesch W, Stauder R, Pfeilstocker M (2014). Azacitidine in 302 patients with WHO-defined acute myeloid leukemia: results from the Austrian Azacitidine Registry of the AGMT-Study Group. Ann Hematol.

[69] Fenaux P, Mufti GJ, Hellstrom-Lindberg E, Santini V, Gattermann N, Germing U (2010). Azacitidine prolongs overall survival compared with conventional care regimens in elderly patients with low bone marrow blast count acute myeloid leukemia. J Clin Oncol.

[70] Issa JP, Garcia-Manero G, Giles FJ, Mannari R, Thomas D, Faderl S (2004). Phase 1 study of low-dose prolonged exposure schedules of the hypomethylating agent 5-aza-2′-deoxycytidine (decitabine) in hematopoietic malignancies. Blood.

[71] Cashen AF, Schiller GJ, O’Donnell MR, DiPersio JF (2010). Multicenter, phase II study of decitabine for the first-line treatment of older patients with acute myeloid leukemia. J Clin Oncol.

[72] Issa JP, Roboz G, Rizzieri D, Jabbour E, Stock W, O’Connell C (2015). Safety and tolerability of guadecitabine (SGI-110) in patients with myelodysplastic syndrome and acute myeloid leukaemia: a multicentre, randomised, dose-escalation phase 1 study. Lancet Oncol.

[73] Paschka P, Schlenk RF, Gaidzik VI, Habdank M, Krönke J, Bullinger L (2010). IDH1 and IDH2 mutations are frequent genetic alterations in acute myeloid leukemia and confer adverse prognosis in cytogenetically normal acute myeloid leukemia with NPM1 mutation without FLT3 internal tandem duplication. J Clin Oncol.

[74] DiNardo CD, Schimmer AD, Yee KWL, Hochhaus A, Kraemer A, Carvajal RD (2016). A phase I study of IDH305 in patients with advanced malignancies Including relapsed/refractory AML and MDS that harbor IDH1^R132^ mutations. Blood.

[75] DiNardo CD, de Botton S, Pollyea DA, Stein EM, Fathi AT, Roboz GJ (2015). Molecular profiling and relationship with clinical response in patients with IDH1 mutation-positive hematologic malignancies receiving AG-120, a first-in-class potent inhibitor of mutant IDH1, in addition to data from the completed dose escalation portion of the phase 1 study. Blood.

[76] Stein EM, DiNardo CD, Pollyea DA, Fathi AT, Roboz GJ, Altman JK (2017). Enasidenib in mutant-IDH2 relapsed or refractory acute myeloid leukemia. Blood.

[77] Porwit-MacDonald A, Ivory K, Wilkinson S, Wheatley K, Wong L, Janossy G (1995). Bcl-2 protein expression in normal human bone marrow precursors and in acute myelogenous leukemia. Leukemia.

[78] Konopleva M, Pollyea DA, Potluri J, Chyla B, Hogdal L, Busman T (2016). Efficacy and biological correlates of response in a phase II study of venetoclax monotherapy in patients with acute myelogenous leukemia. Cancer Discov.

[79] Kontro M, Kumar A, Majumder MM, Eldfors S, Parsons A, Pemovska T (2017). HOX gene expression predicts response to BCL-2 inhibition in acute myeloid leukemia. Leukemia.

[80] Pollyea DA, Dinardo CD, Thirman MJ, Letai A, Wei AH, Jonas BA (2016). Results of a phase 1b study venetoclax with decitabine or azacytidine in untreated acute myeloid leukemia patients ≥65 years ineligible for standard induction therapy. J Clin Oncol.

[81] Lin TL, Strickland SA, Fiedler W, Walter RB, Hou J-Z, Roboz GJ (2016). Phase 1b/2 study of venetoclax with low-dose cytarabine in treatment-naïve patients age ≥65 with acute myelogenous leukemia. J Clin Oncol.

[82] Furqan M, Mukhi N, Lee B, Liu D (2013). Dysregulation of JAK-STAT pathway in hematological malignancies and JAK inhibitors for clinical application. Biomark Res.

[83] Verstovsek S, Odenike O, Singer JW, Granston T, Al-Fayoumi S, Deeg HJ (2016). Phase 1/2 study of pacritinib, a next generation JAK2/FLT3 inhibitor, in myelofibrosis or other myeloid malignancies. J Hematol Oncol.

